# Excitatory Amino Acid Transporters (EAATs): Glutamate Transport and Beyond

**DOI:** 10.3390/ijms20225674

**Published:** 2019-11-13

**Authors:** Simona Magi, Silvia Piccirillo, Salvatore Amoroso, Vincenzo Lariccia

**Affiliations:** Department of Biomedical Sciences and Public Health, School of Medicine, University “Politecnica delle Marche”, Via Tronto 10/A, 60126 Ancona, Italy; s.piccirillo@pm.univpm.it (S.P.); s.amoroso@univpm.it (S.A.); v.lariccia@staff.univpm.it (V.L.)

**Keywords:** antioxidant defenses, excitatory amino acid transporters, glutamate, metabolism

## Abstract

Na^+^-dependent excitatory amino acid transporters (EAATs) are the major transport mechanisms for extracellular glutamate removal in the central nervous system (CNS). The primary function assigned to EAATs is the maintenance of low extracellular glutamate levels, thus allowing glutamate to be used as a signaling molecule in the brain and to avoid excitotoxicity. However, glutamate has other recognized functions. For instance, it is a key anaplerotic substrate for the tricarboxylic acid (TCA) cycle, as it can be converted to α-ketoglutarate by transaminases or glutamate dehydrogenase. Furthermore, glutamate is a precursor of the main antioxidant glutathione, which plays a pivotal role in preventing oxidative cell death. Therefore, glutamate signaling/use is at the crossroad of multiple metabolic pathways and accordingly, it can influence a plethora of cell functions, both in health and disease. Here, we provide an overview of the main functions of glutamate and its transport systems, analyzing its role as a neurotransmitter and at the same time, the possible metabolic fates it can undergo in the intracellular milieu. Specifically, the metabolic role of glutamate and the molecular machinery proposed to metabolically support its transport will be further analyzed.

## 1. Glutamatergic Neurotransmission and Glutamate Transport: An Overview

Glutamate is the primary excitatory neurotransmitter in the mammalian central nervous system (CNS), where it essentially mediates all the rapid excitatory signals. In the brain, glutamate is involved in a plethora of physiologic functions including cognition, memory, learning, nervous system development, cellular migration, cellular differentiation, and neuronal death [1,2]. Glutamate accomplishes these complex roles of both neurotransmitter and neuromodulator through the activation of a diverse set of receptors [2]. Two main classes of receptors have been identified: ionotropic glutamate receptors, which are ligand-gated ion channels producing excitatory glutamate-evoked currents, and metabotropic glutamate receptors, which are G protein-coupled receptors controlling cellular processes via G protein signaling pathways [3]. Based on the agonist selectivity, the members of the first class have been classified as N-methyl-d-aspartate (NMDA), α-amino-3-hydroxy-5-methyl-4-isoxazole propionic acid (AMPA), and kainate (KA) receptors (for a more detailed description of the glutamate receptors see [3]). Thus, glutamate can exert its signaling functions from the extracellular side. An impaired control of the extracellular glutamate concentrations is involved in the pathogenesis of many neurological disorders [4,5,6]. Indeed, it is well established that the overstimulation of NMDA receptors can represent the trigger of multiple neuronal death cascades (=so-called “glutamate excitotoxicity”), leading to apoptosis and necrosis, mainly as a consequence of the massive intracellular Ca^2+^ influx [4,7]. Accordingly, a tight regulation of the extracellular glutamate levels is needed. Considering that there are no known extracellular enzymes that can degrade glutamate [8], the maintenance of low extracellular concentrations relies on the balance of the opposite functions of uptake and release. Glutamate uptake is catalyzed by several transport proteins, however, the term “glutamate transporters” usually refers to the five “Na^+^-dependent high-affinity glutamate transporters”, also known as excitatory amino acid transporters (EAATs) [8]. Other transporter families include the vescicular glutamate transporters (VGLUTs) and the glutamate–cysteine exchanger [9,10,11,12]. In the next section, a detailed description of the EAATs’ family of transporter proteins will be provided.

## 2. Na^+^-Dependent High-Affinity Glutamate Transporters

The “Na^+^-dependent high-affinity glutamate transporters” or “EAATs” belong to the solute carrier 1 (SLC1) family. They are secondary active transporters that take glutamate up into the cell against its concentration gradient. To date, five different glutamate transporter subtypes have been cloned (EAAT1-5). The first glutamate transporter was identified in 1990. It was purified from rat brain membranes by a detergent-based solubilization process followed by conventional chromatographic techniques [8,13,14]. In humans, this transporter subtype is now known as EAAT2, whereas the rodent homologue is termed glutamate transporter-1 (GLT-1, *slc 1a2*). Simultaneously, by using a different approach, another EAAT subtype was identified by a different research team. In 1992, Storck and coworkers purified by chance a 66 kDa hydrophobic glycoprotein, which subsequently resulted in the protein now referred to as EAAT1 in humans and glutamate–aspartate transporter (GLAST, *slc 1a3*) in rodents [8,15]. A third EAAT subtype was identified in the same period by a different research group that isolated this transporter from a rabbit jejunum [8,16]. The human counterpart is now known as EAAT3, while the rodent homologue was termed excitatory amino acid carrier 1 (EAAC1, *scl 1a1*). Later on, two others EAAT subtypes were identified, namely EAAT4 and EAAT5 [17,18]. The five different EAAT subtypes show different patterns of expression (Table 1).

**Figure 1:**
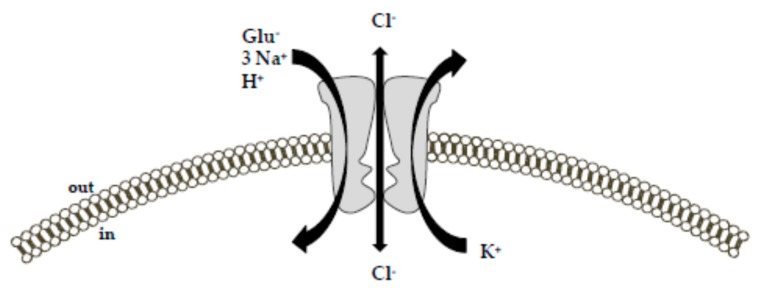
Schematic diagram of the ion-flux coupling stoichiometry for glutamate transporters. The transport of glutamate is coupled with the cotransport of 3 Na^+^, 1 H^+^, and 1 K^+^ ion along their concentration gradient. Additionally, glutamate and Na^+^ activate an uncoupled chloride conductance through the transporter. The picture was adapted from [26] upon written authorization by the editor.

EAAT1 is highly expressed in the neocortex and cerebellum, especially in astrocytes [2,19]; EAAT2 is the main glutamate transporter found in the forebrain; it is abundantly expressed in astrocytes and in a limited extent also on presynaptic nerve terminals [2,20]. While EAAT1 and EAAT2 display a predominant glial expression, EAAT3 appears to be neuron-specific, although EAAT3 expression has been also described in cells of glial origin, i.e., oligodendrocytes [2,21,22,23], rat C6 glioma cells and several human glioma cell models [2,24]. EAAT4 is a neuron-specific glutamate transporter as well, as its expression profile is restricted to Purkinje cells, and EAAT5 is selectively expressed at photoreceptor and bipolar cell terminals in the retina [2,25]. All the EAAT subtypes limit glutamate access to their receptors through a rapid and efficient glutamate removal from the synaptic cleft. Despite some differences in the magnitude of ionic fluxes, they share the same mechanism of transport (Figure 1).

Specifically, the driving force for an effective glutamate uptake is provided by the cotransport of three Na^+^ ions and the countertransport of one K^+^ ion [9,10,27,28]. While the cotransport of Na^+^ occurs simultaneously to the glutamate transport, the countertransport of K^+^ represents an independent step from the glutamate translocation. Together with Na^+^ and glutamate, one H^+^ is also transported. Accordingly, the transport of each glutamate molecule is associated with a net charge movement across the plasma membrane [29]. Consequently, the transmembrane potential can be itself a driving force for the glutamate uptake [10]. In addition to these ion fluxes, glutamate binding to the Na^+^-dependent EAATs generates a thermodynamically uncoupled anion conductance [9,17,26,30,31], which is characterized by the following selectivity sequence: SCN^−^ > ClO^−^_4_ > NO^−^_3_ > I^−^ > Br^−^ > Cl^−^ > F^−^ > gluconate [26,30,32]. Furthermore, a glutamate-independent leak chloride conductance has been also described for these transporters [9,26,30]. The physiological relevance of the glutamate transporter anion conductance is still ill-defined. Based on the available literature, Grewer and colleagues provide an interesting interpretation of this phenomenon [9]. They suggest that the inwardly-directed anion flux may balance the inflow of positive charges (Na^+^) during glutamate translocation. This would help to maintain the membrane potential at a hyperpolarized level that favors Na^+^ entry, and consequently glutamate uptake [9,33]. In this way, neuronal excitability could also be directly modulated. From this perspective, EAATs would serve as glutamate-dependent inhibitory receptors, with the ability to counteract the well-known glutamate excitatory effects [9]. It is interesting to note that the magnitude of the Cl^−^ conductance relative to the coupled transport process varies between the transporter subtypes [34]. In particular, the greatest Cl^−^ conductance magnitude has been described for EAAT4 and EAAT5 subtypes, which, therefore, exhibit a consistent inhibitory function. For EAAT5, the glutamate transporter subtype predominantly expressed in the retina; this function appears plausible. Indeed, in retinal rod bipolar cells, the existence of a pre-synaptic inhibitory glutamate receptor—with pharmacological properties matching those of glutamate transporters rather than those of other chloride channels—has been described in at least two different reports [9,35,36]. This function attributed to the anion flux would be in line with the slow activation of the anion conductance, which delays transporter turnover and, therefore, limits the transport activity [9,37]. EAAT1, EAAT2 and EAAT3 subtypes display a much smaller Cl^−^ conductance with respect to the ion fluxes associated with the transporter function [10,30].

## 3. EAATs and the Maintenance of the Antioxidant Defenses

Although the primary function ascribed to the Na^+^-dependent EAATs is the maintenance of low extracellular glutamate concentrations to avoid cytotoxic effects, several reports have suggested a key role of these transporters in balancing the antioxidant defenses through the provision of intracellular precursors for the tripeptide glutathione (γ-glutamyl-cysteinyl-glycine, GSH) [38]. GSH is the main antioxidant molecule in the brain. It behaves as a radical scavenger without any enzymatic action; it can also serve as an electron donor for the reduction of peroxides by glutathione peroxidase and can be used in detoxification processes by gluthatione-s-transferases [38]. The product of the GSH oxidation is the glutathione disulfide (GSSG). GSH consumption requires a resynthesis through a two steps process based on ATP-driven enzymatic reactions in the presence of glutamate, cysteine and glycine as substrates, with cysteine availability being the rate-limiting factor in the GSH synthesis [39]. Different transport systems taking up cysteine have been identified in astrocytes, including the Xc–system, which transports cysteine in exchange of intracellular glutamate with a 1:1 ratio in physiological conditions [38,40]; a system depending on the γ-GT activity [38,41] and the Na^+^-dependent EAATs [38,42,43]. The role of EAATs in mediating the substrates’ uptake for the synthesis of GSH has recently gained much attention. In principle, when these systems were identified, a functional relationship between EAATs and the Xc–system was hypothesized. It was suggested that EAATs-transported glutamate was necessary to support the activity of the Xc–system, which operates as an exchanger. Subsequent in vivo studies established the inconsistency of this hypothesis, since in mice lacking the Xc system no GSH depletion was observed [38,44], indicating a complementary role for this exchange system. Later, a critical role in this setting was established for EAATs, and, in particular, the high cysteine affinity of EAAC1/EAAT3 in cultured neurons was pointed out by several reports showing that (1) cysteine uptake is Na^+-^dependent, (2) EAATs blockers exert an inhibitory effect on cysteine uptake, (3) in the presence of extracellular glutamate and aspartate, cysteine uptake is blocked, and (4) EAATs inhibitors induce an intracellular GSH depletion and increase the neuronal susceptibility to oxidative stress [39,45,46]. Furthermore, a GSH deficiency in retinal glial cells has been observed in mice lacking glial EAATs [38,47]. In the light of this role described for the EAATs (in particular for EAAC1/EAAT3), the consequences of its specific blockade have been further investigated in in vivo models. In particular, EAAC1-null (Slc1a1–/–) mice show a significant reduction in neuronal GSH levels, accompanied by a parallel increase in oxidant levels, leading to a greater susceptibility to oxidative damage. Noteworthily, all these changes are counteracted by the cysteine precursor N-acetylcysteine, disclosing a main role for EAAC1 in the cysteine uptake process [48]. Furthermore, EAAC1^–^/^–^ mice show a rapid age-dependent loss of dopaminergic neurons in the substantia nigra pars compacta. Neuronal loss is accompanied by increased nitrotyrosine formation, nitrosylated α-synuclein, and microglial activation. The administration of N-acetylcysteine significantly reverses these changes, confirming the critical role exerted by EAAC1 in promoting an overall antioxidant status and pointing out that the oxidative stress may represent an upstream event of the neurodegenerative processes [49]. The role of EAAC1 in preserving the antioxidant defenses of the cells has also been investigated in ischemic settings. In a murine model of focal brain ischemia, EAAC1 knockout reduces brain tolerance to focal ischemia [50]; interestingly, the intracellular GSH levels are not significantly different in control and knockout mice, leading to the concept that EAAC1 absence could have a negative impact on more than a single cellular function. A possible explanation regarding the increased neuronal susceptibility to the ischemic challenge may also rely on the reduced intracellular availability of glutamate as a metabolic substrate rather than on a lack of the antioxidant defenses. The role of EAATs and glutamate in the cellular bioenergetics will be further discussed in the following section.

## 4. Metabolic Role of Glutamate and its Transport Systems

Since the 1980s, research has mostly focused on glutamate as the main excitatory neurotransmitter in the mammalian brain. However, it cannot be overlooked that glutamate is an amino acid, and as such, it can subserve several other functions within a cell. High concentrations of glutamate in the brain were first identified in the 1930s, and considering the high levels observed within cytosolic and mitochondrial compartments, its important metabolic role was immediately recognized. Glutamate has an important role in cell bioenergetics: through its conversion to α-ketoglutarate, glutamate can enter the tricarboxylic acid (TCA) cycle, thus participating as an anaplerotic substrate in supporting mitochondrial respiration. This function is relevant in such organs (i.e., brain and heart) that are characterized by a high metabolic rate. As interestingly reported by McKenna [2,51], glutamate per se can activate glial and neuronal energy metabolism [2,52,53,54]. Glial cells have a major role in clearing glutamate from the synaptic space. Once in the cytoplasm, glutamate can be used in different pathways, depending on its own extracellular concentration [2]. For instance, when extracellular glutamate concentrations are rather low, the taken-up glutamate is rapidly converted to glutamine. On the contrary, higher extracellular glutamate concentrations imply its metabolic use within the TCA cycle [51]. Although it is well established that neuronal energy production mainly relies on glucose oxidation, several reports have indicated that synaptic terminals and primary cultures of neurons can also use both glutamine and glutamate for energy supply [2,55,56,57]. For instance, Divakaruni et al. [58] revisited the consolidated concept that neurons depend on glucose to sustain their mitochondrial metabolism. By performing ^13^C tracer analyses, they determined the fate of ^13^C-labeled nutrients by following the labeled carbons through the metabolic network. Even in glucose-rich conditions, they interestingly found that neurons can use alternative nutrients for mitochondrial energy production (i.e., leucine and β-hydroxybutyrate). Once they established that neurons can use alternative nutrients to fuel mitochondrial metabolism, the authors investigated how neurons respond when the use of glucose is largely precluded, for instance, by inhibiting pyruvate entry into the mitochondria. Pyruvate, the major downstream product of glucose, is transported into the mitochondria via the mitochondrial pyruvate carrier (MPC): its inhibition excludes glucose and any other glycolytic carbon sources, including lactate, as substrates to boost mitochondrial metabolism. Strikingly, MPC inhibition did not affect mitochondrial energy production since neurons switch to glutamate oxidation as an alternative to glucose [58,59]. Broadening these results to more complex systems led to the concept that cell bioenergetics and neurotransmission are closely related processes, and that glutamate may be the link between them [2,51]. The findings recently observed by our research group fit into this scenario. In 2012 we reported that, under physiological conditions, in purified rat brain and heart mitochondria, glutamate—at the concentration of 1 mM—can elicit ATP de novo synthesis [60]. Our studies unravel two main novelties. Firstly, in the specific analyzed experimental setting, where glutamate is the main source of energy, glutamate entry into the mitochondria completely relies on EAATs activity, rather than on the well-established aspartate/glutamate carriers activity (AGCs [61,62]). This observation lends support to the emerging concept considering EAATs more than mere “glutamate sink”. Instead, they can subserve different functions, ranging from maintenance of antioxidant defenses, protection from excitotoxicity, intracellular signal transduction [63], and cell energy metabolism fueling. On the other hand, a role for GLAST in the malate-aspartate shuttle was already observed by Ralphe et al. in rat cardiac mitochondria [64,65]. The metabolic role exerted by EAATs seems to be highly specific. To the best of our knowledge, glutamate influx into mitochondria is specifically mediated by EAAT3/EAAC1 [60].

**Figure 2:**
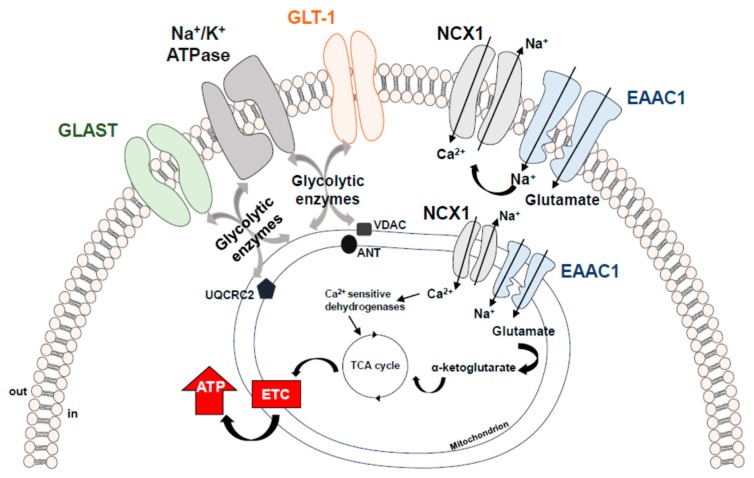
Schematic representation of the EAATs-including macromolecular complexes. EAATs compartmentalization with Na^+^/K^+^-ATPase and NCX1 ensures an efficient glutamate uptake through the maintenance of ion gradients. Compelling evidence supports the concept that the increased Na^+^ influx generated by the EAAC1 forces NCX1 to work on the reverse mode, thereby activating a virtuous cycle that could induce a slight but significant local increase in Ca^2+^ levels and stimulate the activity of the Ca^2+^-sensitive mitochondrial dehydrogenases, fueling ATP synthesis [2]. EAATs local assembly with the “energy producing machinery” further optimizes ATP production, creating a local proteins pool able to quickly support and adjust energy demand. ANT = Adenine Nucleotide Translocator; EAAC1 = Excitatory Amino Acid Carrier1; GLAST = Glutamate Aspartate Transporter; GLT-1 = Glutamate Transporter-1; NCX1 = Na^+^/Ca^2+^ exchanger1; UQCRC2 = Ubiquinol Cytochrome c Oxidoreductase Subunit Core 2; VDAC = Voltage-Dependent Anion Channel.

Secondly, we intriguingly report that, in mitochondria, EAAT3/EAAC1 by itself is not able to ensure effective glutamate uptake to drive ATP synthesis, its activity needs to be sustained by another transporter, the Na^+^/Ca^2+^ exchanger (NCX). NCX is one of the main regulators of the intracellular Ca^2+^ homeostasis. NCX catalyzes the bidirectional and electrogenic exchange of 3 Na^+^ and 1 Ca^2+^ ions across the plasma membrane, operating either in Ca^2+^-efflux/Na^+^-influx mode (forward mode) or Ca^2+^-influx/Na^+^-efflux mode (reverse mode) [66,67,68]. NCX belongs to a multigene family (*Slc8a1-3*) encoding three different isoforms—NCX1, NCX2, and NCX3—which display a tissue-specific distribution [69,70]. EAAT3/EAAC1 establishes a physical and functional interaction with NCX1, making up a macromolecular complex able to modulate the glutamatergic machinery towards energy production. Subsequent studies allowed us to expand our knowledge on such a specific interaction. We found that (1) the macromolecular complex made up by EAAT3/EAAC1 and NCX1 also exists within the plasma membrane of glial, neuronal and cardiac cells and that it is essential to mediate glutamate-driven ATP synthesis [71]; (2) such a complex can be a complementary route for substrates utilization under energy-compromised conditions (i.e., hypoxic settings) [72,73]. Both in cardiac and neuronal hypoxic settings, the EAAT3/EAAC1-NCX1 macromolecular complex drives glutamate utilization towards the synthesis of ATP, ultimately improving cell survival [72,73] (for a more detailed description of the EAAT3/EAAC1-NCX1-driven glutamate metabolic utilization see [2]). The co-assembly of functionally related proteins within macromolecular complexes can positively influence the specificity and efficiency of biological processes taking place within a specific milieu. In the case of the EAAT3/EAAC1-NCX1 macromolecular complex, its main function is to ensure an optimal glutamate utilization, with special regard to such conditions potentially requiring a metabolic enhancement. In line with our reports, other studies have claimed that glutamate transporters may be associated with the energy producing cellular machinery for glycolysis and oxidative phosphorylation [74] in order to spatially and functionally optimize energy demand. Undoubtedly, the maintenance of low glutamate concentrations in the synaptic space is a high energy-consuming process [75]. GLT-1 and GLAST were found to be physically—and most probably also functionally—linked to Na^+^/K^+^ ATPase, to mitochondrial matrix proteins specifically, Voltage-dependent anion channel (VDAC), ubiquinol cytochrome c oxidoreductase subunit core 2 (UQRC2) and adenine nucleotide translocator (ANT) and to several glycolytic enzymes, i.e., hexokinase 1 and glyceraldehyde 3-phosphate dehydrogenase (GAPDH) [74,75,76]. Although it has not been fully proven, intermediary proteins may be responsible for the link between EAATs and the above-mentioned proteins in each specific setting. This supramolecular functional assembly of EAATs with either metabolic proteins or signaling/transporting molecules has been designated as “glutamosome” [74]. Within the “glutamosome” EAATs are associated with proteins that ensure a proper glutamate uptake through the maintenance of Na^+^ gradient, and, at the same time, such an assembly provides an energetically privileged route to productively use glutamate as an ATP source, making up an efficient machinery supporting specific energy needs [74] (Figure 2).

## 5. Conclusions

Na^+^-dependent EAATs are the principal transport systems for extracellular glutamate clearance within the CNS. In this view, EAATs have been considered for a long time as mere “glutamate sink”. However, considering the diverse functions exerted by glutamate within a cell, EAATs’ role has generally been reconsidered. For instance, beyond a neurotransmitter, glutamate can serve as a precursor of the main antioxidant GSH and can be a critical anaplerotic substrate for the TCA cycle. Accordingly, EAATs may influence several cell functions, including not only the neurotransmission and the prevention of the excitotoxicity, but also the overall cellular redox state and energy metabolism. Several reports have highlighted that to efficiently accomplish such delicate functions, EAATs co-assembly with specific proteins to form functional complexes aimed at adequately controlling glutamate utilization in time and space. Such assembly has been interestingly dubbed as “glutamosome”. Within the “glutamosome” glutamate machinery ensures a proper utilization of this substrate, making this complex an interesting target that could be modulated in such conditions requiring a specific control of both oxidative stress and metabolic dysfunctions (i.e., ischemia/reperfusion settings and neurodegenerative diseases). Thus, an interesting approach could be the enhancement of the cross-talk between the component of the “glutamosome”, and in this view, further investigations and functional studies will be needed to test its actual therapeutic potential.

## Figures and Tables

**Table 1:** Na^+^-dependent excitatory amino acid transporters (EAATs): glutamate–aspartate transporter (GLAST), glutamate transporter-1 (GLT-1), excitatory amino acid carrier1 (EAAC1).

Glutamate Transporters Subtype	Rodent Homologue	Cell Type	DISTRIBUTION
EAAT1	GLAST	Astrocytes, oligodendrocytes [2,19]	Cerebellum, cortex, spinal cord
EAAT2	GLT-1	Astrocytes [2,20]	Through the brain and spinal cord
EAAT3	EAAC1	Mostly neurons. Also found in cells of glial origin (i.e., oligodendrocytes, glioma cells) [2,21,22,23]	Hippocampus, striatum, cerebellum
EAAT4	EAAT4	Purkinje cells [2,24]	Cerebellum
EAAT5	EAAT5	Photoreceptor and bipolar cells [2,25]	Retina

## Abbreviations

AGCs	Aspartate/Glutamate Carriers
ANT	Adenine Nucleotide Translocator
AMPA	α-Amino-3-hydroxy-5-Methyl-4-Isoxazole Propionic acid
EAAC1	Excitatory Amino Acid Carrier 1
EAATs	Excitatory Amino Acid Transporters
GAPDH	Glyceraldehyde 3-Phosphate Dehydrogenase
GLAST	Glutamate Aspartate Transporter
GLT-1	Glutamate Transporter-1
GSH	γ-Glutamyl-Cysteinyl-Glycine
GSSG	Glutathione Disulfide
KA	Kainate
MPC	Mitochondrial Pyruvate Carrier
NMDA	N-Methyl-D-Aspartate
TCA	Tricarboxylic Acid
UQRC2	Ubiquinol Cytochrome c Oxidoreductase Subunit Core 2
VDAC	Voltage-Dependent Anion Channel
VGLUTs	Vescicular Glutamate Transporters
